# Glymphatic system impairment in nonathlete older male adults who played contact sports in their youth associated with cognitive decline: A diffusion tensor image analysis along the perivascular space study

**DOI:** 10.3389/fneur.2023.1100736

**Published:** 2023-02-15

**Authors:** Yuichi Morita, Koji Kamagata, Christina Andica, Kaito Takabayashi, Junko Kikuta, Shohei Fujita, Thomas Samoyeau, Wataru Uchida, Yuya Saito, Hiroki Tabata, Hitoshi Naito, Yuki Someya, Hideyoshi Kaga, Yoshifumi Tamura, Mari Miyata, Toshiaki Akashi, Akihiko Wada, Toshiaki Taoka, Shinji Naganawa, Hirotaka Watada, Ryuzo Kawamori, Osamu Abe, Shigeki Aoki

**Affiliations:** ^1^Department of Radiology, Graduate School of Medicine, Juntendo University, Tokyo, Japan; ^2^Department of Radiology, Graduate School of Medicine, The University of Tokyo, Tokyo, Japan; ^3^Faculty of Health Data Science, Juntendo University, Chiba, Japan; ^4^Department of Radiology, Necker Hospital, Paris University, Paris, France; ^5^Sportology Center, Graduate School of Medicine, Juntendo University, Tokyo, Japan; ^6^Department of Metabolism and Endocrinology, Graduate School of Medicine, Juntendo University, Tokyo, Japan; ^7^Graduate School of Health and Sports Science, Juntendo University, Inzai, Chiba, Japan; ^8^Department of Functional Brain Imaging, National Institutes for Quantum and Radiological Science and Technology, Chiba, Japan; ^9^Department of Innovative Biomedical Visualization, Graduate School of Medicine, Nagoya University, Nagoya, Japan; ^10^Department of Radiology, Graduate School of Medicine, Nagoya University, Nagoya, Japan

**Keywords:** repetitive head impacts, contact sports, glymphatic system, DTI-ALPS, diffusion-weighted imaging, cerebrospinal fluid, interstitial fluid

## Abstract

**Background and purpose:**

Exposure to contact sports in youth causes brain health problems later in life. For instance, the repetitive head impacts in contact sports might contribute to glymphatic clearance impairment and cognitive decline. This study aimed to assess the effect of contact sports participation in youth on glymphatic function in old age and the relationship between glymphatic function and cognitive status using the analysis along the perivascular space (ALPS) index.

**Materials and methods:**

A total of 52 Japanese older male subjects were included in the study, including 12 who played heavy-contact sports (mean age, 71.2 years), 15 who played semicontact sports (mean age, 73.1 years), and 25 who played noncontact sports (mean age, 71.3 years) in their youth. All brain diffusion-weighted images (DWIs) of the subjects were acquired using a 3T MRI scanner. The ALPS indices were calculated using a validated semiautomated pipeline. The ALPS indices from the left and right hemispheres were compared between groups using a general linear model, including age and years of education. Furthermore, partial Spearman's rank correlation tests were performed to assess the correlation between the ALPS indices and cognitive scores (Mini-Mental State Examination and the Japanese version of the Montreal Cognitive Assessment [MoCA-J]) after adjusting for age years of education and HbA1c.

**Results:**

The left ALPS index was significantly lower in the heavy-contact and semicontact groups than that in the noncontact group. Although no significant differences were observed in the left ALPS index between the heavy-contact and semicontact groups and in the right ALPS index among groups, a trend toward lower was found in the right ALPS index in individuals with semicontact and heavy-contact compared to the noncontact group. Both sides' ALPS indices were significantly positively correlated with the MoCA-J scores.

**Conclusion:**

The findings indicated the potential adverse effect of contact sports experience in youth on the glymphatic system function in old age associated with cognitive decline.

## 1. Introduction

Contact sports, such as American football and soccer, involve physical contact between players, and these affect brain health (1). Contact sports have been linked to neurocognitive changes due to repetitive head impacts (RHIs) (1–3). RHIs refer not only to mild traumatic brain injury and concussion but also to asymptomatic subconcussive trauma (4, 5). RHIs in contact sports are quite frequent, with up to 3–70 head impacts per game that players were exposed to, depending on the sport (6, 7). Previous animal studies have suggested that RHIs lead to neuroinflammation, synaptic changes, and glymphatic system dysfunction (8, 9). Recently, glymphatic dysfunction has been considered one of the main causes of cognitive decline due to the accumulation of brain waste products (10, 11). However, its involvement in older adults with contact sports participation in their youth is not yet fully understood.

The glymphatic system is a brain waste clearance system (11, 12). The underlying hypothesis is that CSF flows into the brain through the perivascular space around the arteries and enters the brain parenchyma through aquaporin-4 (AQP4) channels in the astrocyte endfeet. Then, CSF influx into the brain parenchyma promotes interstitial fluid (ISF). Finally, brain metabolic waste products are washed out through the perivascular space around the veins (11–13). Previous animal studies have shown reduced clearance of intrathecally injected gadolinium contrast agents and fluorescent tracers in the brain of RHI-injured rodents (8, 12, 14). However, the tracer-based method used to assess the glymphatic system is invasive and thus not suitable for human studies.

Taoka et al. proposed diffusion tensor imaging analysis along the perivascular space (DTI-ALPS) (15). The analysis ALPS index is a potential indirect noninvasive indicator of the glymphatic system in humans by estimating the diffusivity of the perivascular space along the medullary veins at the level of the lateral ventricular body. Zhang et al. reported a strong correlation between the ALPS index and glymphatic function assessed using the intrathecal injection of gadolinium contrast agents in human brains (16). Furthermore, a reduced ALPS index has been correlated with cognitive decline in older adults and patients with Alzheimer's or Parkinson's disease (15, 17, 18).

This study aimed to evaluate the effect of contact sports practice in youth on the glymphatic function in old age using the ALPS index and to study the relationship between glymphatic function and cognitive status.

## 2. Methods

### 2.1. Study participants

A total of 52 community-dwelling older adults (66–83 years old) enrolled in the Healthy Brain Project by the Sportology Center of Juntendo University in Tokyo, Japan, from 2017 to 2018 (19), were included in this study. This study was approved by our Institutional Review Board. Written informed consent was obtained from all participants before evaluation.

The inclusion criteria included nonathlete subjects with data of sports experience in their teenage and 20s, diffusion-weighted images (DWIs), cognitive scores (Mini-Mental State Examination [MMSE], and the Japanese version of the Montreal Cognitive Assessment [MoCA-J]). The exclusion criteria were subjects with cerebrovascular disease, severe cognitive decline (MMSE <23), and a history of severe traumatic brain injury or psychiatric or neurological disorders.

Contact sports are sports in which players collide (20, 21). To further understand the influence of the intensity of the collision in contact sports on the glymphatic system, study participants were categorized into heavy-contact and semicontact sports groups (20, 21). Heavy-contact sports are sports with intense physical contact where the player is allowed to continuously and intentionally strike or tackle the opponent (20, 21). Meanwhile, semicontact sports are sports with occasional physical contact, and intentional hitting or tackling is prohibited (20, 21). For comparison, we also included age-matched individuals with experience in noncontact sports in their youth (noncontact group). Only male participants were included in this study to minimize the influence of sex differences. Noncontact sports are those with little or no physical contact (20, 21).

### 2.2. Study participants' characteristics

This study included data obtained from 52 older Japanese male adults, of whom 12 (71.2 ± 5.2 years) had experience with heavy-contact sports in their youth; 15 (73.1 ± 5.9 years) with semicontact sports; and age-matched 25 (71.3 ± 4.4 years) with noncontact sports. Demographic and clinical characteristics of the study participants in the noncontact, semicontact, and heavy-contact groups are summarized in Table 1.

**Table 1:** Demographic and clinical characteristics of the study participants.

	**Noncontact Group**	**Semicontact Group**	**Heavy-Contact Group**	***p-*Values**	**Semicontact versus Heavy-Contact**	**Noncontact versus Semicontact**	**Noncontact versus Heavy-Contact**
Number of subjects	25	15	12				
Age (years)	71.3 ± 4.4	73.1 ± 5.9	71.2± 5.2	0.67^b^			
Years of education (years)	15.4 ± 1.7	14.9 ± 1.8	14.1 ± 2.5	0.19^b^			
BMI (kg/m^2^)	23.5 ± 2.9	23.9 ± 1.9	22.7 ± 3.7	0.27^b^			
Sport experience during youth (years)	5.0 ± 4.1	5.4 ± 2.4	8.0 ± 2.3	0.005^b^	0.06^c^	0.21^c^	0.002^c^
Handedness (right, left, ambidextrous)	23, 0, 2	18, 0, 0	10, 1, 1	0.23^a^			
MMSE	27.6 ± 1.5	28.6 ± 1.3	27.9 ± 1.7	0.17^b^			
MoCA-J	25.4 ± 2.6	25.0 ± 3.0	24.5 ± 2.5	0.16^b^			
Smoking history (Brinkman index)	272.2 ± 326.8	767.7 ± 803.5	592.6 ± 580	0.15^b^			
Alcohol consumption (g/day)	13.0 ± 15.6	33.2 ± 36.6	33.3 ± 24.7	0.10^b^			
Systolic blood pressure (mmHg)	137.2 ± 14.8	136.5 ± 20.6	137.9 ± 12.7	0.91^b^			
Diastolic blood pressure (mmHg)	87.3 ± 8.1	88.3 ± 12.7	88.4 ± 8.6	0.77^b^			
HbA1c (%)	6.0 ± 0.8	5.9 ± 0.6	5.5 ± 0.3	0.07^b^			
Intake of carbonhydrates (g/day)	262.2 ± 63.6	235.8 ± 76.9	208.7± 63.9	0.054^b^			
Current exercise time (Mets/week)	14.2 ± 10.5	9.1 ± 10.8	9.6± 17.0	0.38^b^			
High-density lipoprotein cholesterol (mg/dL)	59.5 ± 12.5	58,2 ± 10.6	57.0± 16.9	0.86^b^			
Low-density lipoprotein cholesterol (mg/dL)	116.6 ± 25.5	113.6 ± 39.5	107.5 ± 31.9	0.37^b^			
Fazekas grade:							
Periventricular white matter (grade: 0/1/2)	(1, 21, 4)	(0, 10, 5)	(0, 8, 4)	0.54^a^			
Deep and subcortical white matter (grade: 0/1/2/3)	(0, 22, 4, 0)	(0, 8, 5, 2)	(0, 9,3, 0)	0.12^a^			

Data are expressed as mean ± standard deviation unless otherwise specified. Statistical analyses are performed using ^a^Fisher's exact test, ^b^Kruskal–Wallis test, and ^c^Mann–Whitney U-test. BMI, body mass index; MMSE, Mini-Mental State Examination; MoCA-J, Japanese version of the Montreal Cognitive Assessment; HbA1c, hemoglobin A1c.

The heavy-contact and semicontact groups had significantly more years of sports experience than the noncontact group. No significant differences were observed in age, handedness, body mass index, years of education, systolic and diastolic blood pressures, high- and low-density lipoprotein cholesterol levels, HbA1c level, the Brinkman index, daily alcohol consumption, the Fazekas grade, the MMSE score, and the MoCA-J score among the three groups.

The number of years of sports experience was 8.0, 5.4, and 5.0 years in the heavy-contact, semicontact, and noncontact groups, respectively. The subjects were not interviewed about their participation as varsity athletes. Meanwhile, those in the noncontact group had no contact sports experience. Data regarding the heavy-, semi-, and noncontact sports played by the study participants in their youth are shown in Table 2.

**Table 2:** Categorization of sports.

	**Sports**	**Number of participants**
Heavy-contact sports	Rugby, judo, karate, boxing, kendo, and wrestling, soccer	12
Semicontact sports	baseball, basketball, and handball	15
Noncontact sports	Tennis, table tennis, track and field, skiing, archery, and orienteering	25

### 2.3. MRI data acquisition

All DWIs were acquired on a MAGNETOM Prisma 3T MRI scanner (Siemens, Erlangen, Germany) with a 64-channel head coil. Whole brain DWIs were acquired using multislice echo-planar imaging along 64 diffusion gradient directions in the anterior-posterior direction at a b-value = 1,000 s/mm^2^ with one nondiffusion-weighted (*b* = 0) volume using the following parameters: TR/TE = 3,300/70 ms, matrix size = 130 × 130, resolution = 1.8 mm × 1.8 mm, slice thickness = 1.8 mm, FOV = 229 mm × 229 mm, and acquisition time = 7 min 29 s. Furthermore, standard and reverse phase-encoded blipped images without diffusion weighting (blip-up and blip-down) were also acquired to correct magnetic susceptibility-induced distortions related to EPI acquisition (22).

### 2.4. DWI data processing

All DWIs were checked visually for severe artifacts in the axial, coronal, and sagittal planes. Noise and artifacts in DWIs were corrected using Marcenko–Pastur principal component analysis denoising (23) and degibbs correction using MRtrix (https://www.mrtrix.org/) (24). Furthermore, the geometric distortions caused by eddy currents and motion-induced susceptibility (25) were corrected using the EDDY and TOPUP toolboxes, parts of the FMRIB Software Library (FSL; https://fsl.fmrib.ox.ac.uk/fsl/fslwiki) version 6.04 (26). We obtained diffusivity maps for each study participant in the *x*-axis (right–left, Dxx), *y*-axis (anterior–posterior, Dyy), and *z*-axis (inferior–superior, Dzz) directions. We also generated fractional anisotropy (FA) maps for all subjects and registered them in the FMRIB58_FA standard space (https://fsl.fmrib.ox.ac.uk/fsl/fslwiki/FMRI*B5*8_FA) using FSL's linear image registration tool (http://www.fmrib.ox.ac.uk/fsl/fslwiki/FLIRT) and nonlinear registration tool (http://fsl.fmrib.ox.ac.uk/fsl~/fslwiki/FNIRT).

### 2.5. ALPS index calculation

**Figure 1:**
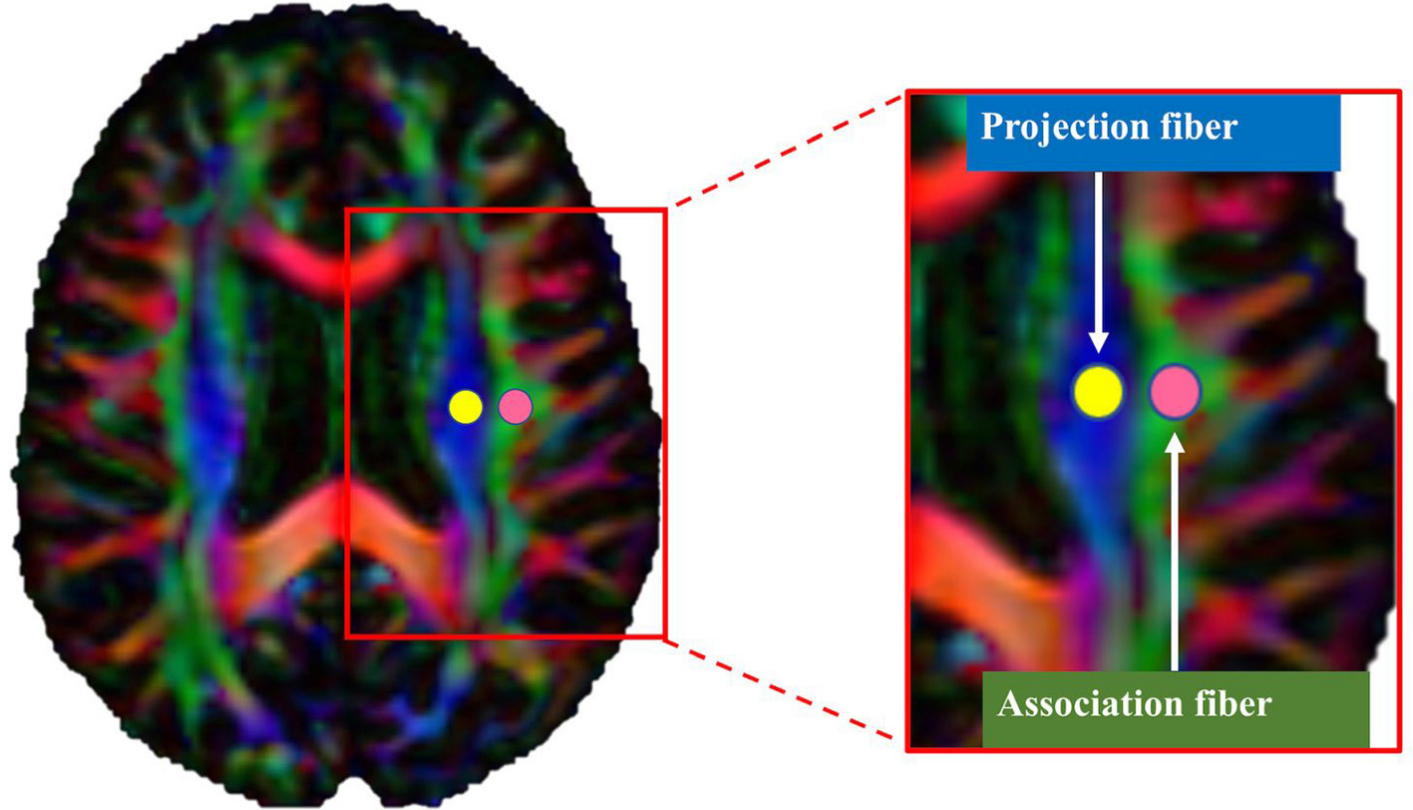
Region of interest (ROI) placement for calculating the along the perivascular space index. Spherical ROIs measuring 5 mm in diameter were placed in the areas of the projection (yellow) and association (pink) fibers.

The ALPS index was calculated using a validated semiautomated pipeline (27). One subject (a 68-year-old male control subject) with the smallest degree of warping was selected to place the region of interest (ROI). Using the color FA map of this subject, spherical ROIs measuring 5 mm in diameter were placed in the projection and association regions at the level of the lateral ventricular body in the left and right hemispheres (Figure 1) (27). Then, the obtained ROIs were registered to the same FA template. Finally, the ROI's position on the FA images of each subject was manually checked. Since all ROIs were correctly positioned, no manual correction was performed.

The values of *x*-, *y*-, and *z*-axis diffusivities in the ROIs were calculated for each individual. In the plane of the lateral ventricle, medullary vessels run in the right-left (*x*-axis) direction. Therefore, perivascular spaces are oriented along the *x*-axis. In this specific anatomical region, main white matter fibers run orthogonally to the *x*-axis (i.e., to the perivascular space direction), with projection fibers along the *z*-axis and association fibers along the *y*-axis. The ALPS index was then calculated as the ratio of the average *x*-axis diffusivity of the projection region (Dxxproj) and the average *x*-axis diffusivity of the association region (Dxxassoc) to the average *y*-axis diffusivity of the projection region (Dyyproj) and the average *z*-axis diffusivity of the association region (Dzzassoc), as follows:


(1) $$ALPS\;index=\frac{Mean\;(D_{xx,proj},\;D_{xx,assoc})}{Mean\;(D_{yy,proj},\;D_{zz,assoc})}$$

An ALPS index close to 1.0 indicates less diffusivity along the space around the perivascular space, while higher values indicate increased diffusivity. The left and right ALPS indices were calculated.

### 2.6. Statistical analysis

Data normality was assessed using the Shapiro–Wilk test. Categorical and continuous data of participants' characteristics were compared using Fisher's exact test and the Kruskal–Wallis test, respectively. Left and right ALPS indices were compared among the heavy-contact, semicontact, and noncontact groups using a general linear model (GLM) analysis, including age and years of education and Hemoglobin A1c (HbA1c). Although no significant difference was found in HbA1c values among groups, the values were higher in the noncontact and semicontact groups. Furthermore, diabetes mellitus has been reported to cause changes in the function of the glymphatic system (28). Therefore, in this study, we decided to include HbA1c as a confounding factor. The heavy-contact and semicontact groups had significantly longer years of sports experience than the noncontact group. Thus, as additional analyses, group comparisons and partial correlation analyses were performed, including years of sports experience as a confounding factor. Pairwise comparisons of groups were then performed using the Tukey–Kramer *post hoc* tests. In addition, we used Cohen's *d* to estimate the effect size among the three groups. Cohen's *d* of 0.1–0.3, 0.3–0.5, 0.5–0.7, and >0.7 were considered as small, medium, large, and very large effect sizes, respectively (29). The associations between the left or right ALPS indices and the MMSE or MoCA-J scores after adjusting for age years of education and HbA1c were evaluated in the semicontact and heavy-contact groups combined using partial Spearman's rank correlation tests. In all analyses, a *p* < 0.05 was considered statistically significant. Due to the exploratory nature of this study, we did not perform corrections for multiple comparisons. Statistical analysis was performed using R version 4.12 (The R Foundation for Statistical Computing, Vienna, Austria).

## 3. Results

### 3.1. Between-group differences

**Figure 2:**
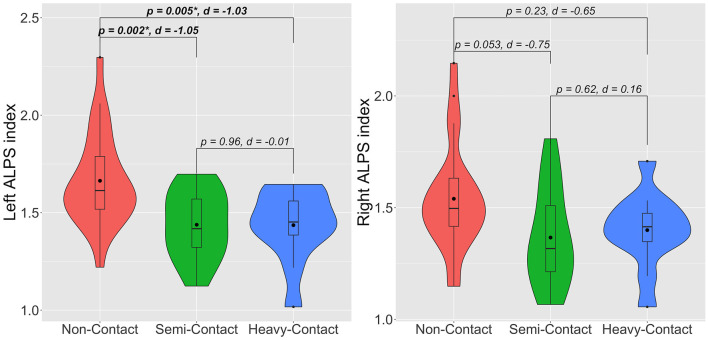
Violin plots of left and right ALPS indices for noncontact (red), semicontact (green), and heavy-contact (blue) groups. Boxes indicate the interquartile range (75th [upper horizontal line] and 25th [lower horizontal line]), mean (bold black line), and median (black dot). Upper whiskers indicate the maximum value of the variable at a distance of 1.5 times the quartile range from the 75th percentile value. Lower whiskers indicate the distance to the 25th percentile value. Small dots indicate an outlier. Surrounding the boxes (shaded area) on each side is a rotated kernel density plot. The figure shows *p*-values after adjusting for age, years of education, HbA1c, and Cohen's *d*. *Significant to *P* < 0.05. ALPS, analysis along the perivascular space.

After adjusting for age years of education and HbA1c, the heavy-contact (*p* = 0.005, Cohen's *d* = −1.03) and semicontact (*p* = 0.002, Cohen's *d* = −1.05*)* groups had significantly lower left ALPS index with very large effect sizes than the noncontact group (Figure 2). Meanwhile, a trend toward the lower right ALPS index was demonstrated in the heavy-contact (*p* = 0.23, Cohen's *d* = −0.65) and semicontact (*p* = 0.053, Cohen's *d* = −0.75) groups with very large and large effect sizes, respectively, compared to the noncontact group. As expected, effect sizes were larger in the heavy-contact group than in the semicontact group. No significant differences in left or right ALPS indices were observed between the heavy-contact and semicontact groups.

In the additional group comparison analysis, consistent results were obtained after adjusting age, years of education, HbA1c, and years of sports experience. The heavy-contact (*p* = 0.03) and semicontact (*p* = *0.0*03) groups had significantly lower left ALPS index than the noncontact group. No significant differences were observed in the left ALPS index between the heavy-contact and semicontact groups and in the right ALPS index among groups.

### 3.2. Associations between cognitive performance and ALPS index

**Figure 3:**
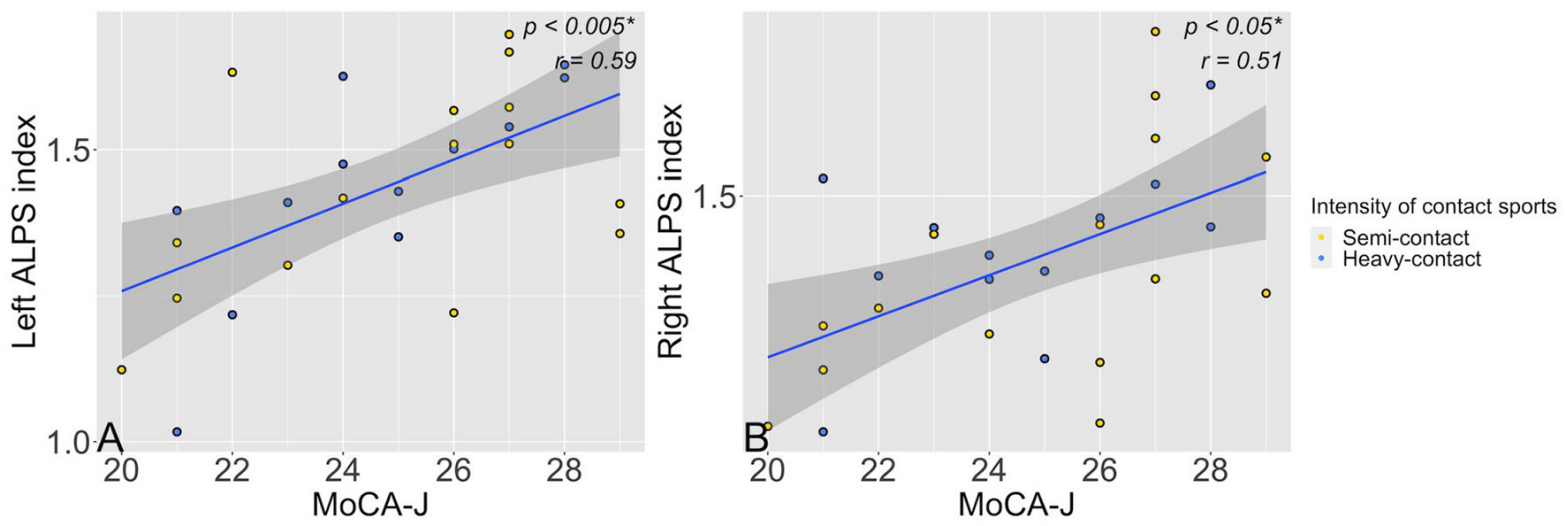
Scatter plots show a significant (*p* < 0.05) association between left **(A)** or right **(B)** ALPS along the perivascular space ALPS indices and MoCA-J scores in the semicontact (yellow dots) and heavy-contact groups (blue dots) combined. *Significant to *P* < 0.05. ALPS, analysis along the perivascular space; MoCA-J, Japanese version of the Montreal Cognitive Assessment.

Lower left (*p* = 0.003, *r* = 0.59) or right (*p* = 0.01, *r* = 0.51) ALPS indices in heavy-contact and semicontact groups combined were significantly correlated with worse MoCA-J scores after adjusting for age, years of education, and HbA1c (Figure 3). However, no significant correlation was observed between MMSE scores and left (*p* = 0.74, *r* = 0.07) or right ALPS (*p* = 0.56, *r* = 0.13) indices.

The additional partial correlation analysis showed similar results when adjusted for age, years of education, HbA1c, and years of sports experience. The MoCA-J and ALPS indices showed significant correlations on both sides: left (p = 0.003, r = 0.59) and right (p = 0.009, r = 0.53), whereas MMSE and ALPS indices showed no significant correlation: left (p = 0.78, r = 0.06) and right (p = 0.39, r = 0.19).

## 4. Discussion

We evaluated the effects of youth contact sports experiences on glymphatic system function in community-dwelling older adult males (66–83 years old) using the DTI-ALPS index. The study findings showed a significantly lower left ALPS index and a trend toward a lower right ALPS index in individuals with semicontact and heavy-contact sports experience in their youth than in those with noncontact sports experience, which are likely to be related to the impairment of glymphatic function (Figure 2). As expected, larger effect sizes were observed in individuals with heavy-contact sports experience than in those with semicontact sports experience, which indicates a more severe decrease in glymphatic function in those with heavy-contact sports experience. Furthermore, the partial correlation analyses showed significant associations between lower left or right ALPS indices and lower MoCA-J scores (Figure 3).

The lower ALPS index reflects decreased diffusivity along the perivascular space of the deep medullary vein. Previous studies have reported that RHIs and mild head impacts cause glymphatic dysfunction in animal experiments using gadolinium contrast agents and fluorescent tracers (30, 31). Ren et al. found decreased AQP4 expression in mice's perivascular space in the cerebral cortex and striatum after mild head impacts (32). AQP4 plays an important role in facilitating the CSF and ISF exchange (10). Therefore, reduced AQP4 expression due to RHIs might have also impaired CSF-ISF drainage toward the perivenous space leading to a reduced ALPS index. Furthermore, the risk of RHIs is increased in high-intensity contact sports, such as rugby (33, 34). Given that larger effect sizes were observed in the heavy-contact group than in the semicontact group, we also speculate that higher intensity of contact sports might cause more severe impairment of the glymphatic system.

Although the fluid dynamics of CSF and ISF have not been fully clarified, CSF/ISF dynamics impairment has been in many diseases, including Alzheimer's disease, Parkinson's disease, and stroke (15–17). Taoka et al. proposed the concept of central nervous system (CNS) interstitial fluidopathy, which would group pathologies associated with abnormal neurofluid dynamics (35, 36). Arterial pulsatilities are an important driving force of CSF/ISF flow (37). Several studies have also demonstrated the associations between RHIs and vascular pathology, which likely contributes to long-term detrimental effects on cerebrovascular functions (38, 39) and ISF-CSF exchange (40). Taken together, the results of this study also indicate that CNS interstitial fluidopathy might be related to contact sports due to RHIs.

Interestingly, the semicontact and heavy-contact groups showed a significantly reduced left ALPS index compared with the noncontact group. In contrast, there was only a trend toward a lower right ALPS index. One possible reason for the left–right difference observed in this study is that the left cerebral hemisphere may be more vulnerable to head impact than the right hemisphere. These findings are consistent with previous studies that examined subjects experiencing mild traumatic brain injury and reported white matter hypoperfusion, microstructural changes, and cortical thinning predominantly in the left hemisphere (41–43). In addition, as mentioned above, RHIs can cause glymphatic system dysfunction due to pathological changes in the cerebral arteries. The difference in the bifurcation of the right and left carotid arteries suggests that the left carotid is more directly susceptible to strong pulse pressure from the aortic arch and is more likely to experience severe plaque formation and intimal damage (27, 44). Thus, this may have led to increased glymphatic dysfunction in the left cerebral hemisphere.

The study findings showed a significant relationship between semicontact and heavy-contact sports-related glymphatic system dysfunction and MoCA-J scores after adjusting for age, years of education, and HbA1c. This further supports the negative effect of RHIs in youth on cognitive function later in life, possibly due to glymphatic dysfunction, using the ALPS index as an objective imaging indicator of cognitive function. In line with the study results, significant correlations were observed between lower ALPS index and poorer cognitive performance in healthy older adults and patients with Alzheimer's disease or cardiovascular disease (15, 16), and impaired glymphatic function was also observed. In this study, a significant positive correlation was observed between the ALPS index and the MoCA-J score, whereas an insignificant correlation was observed between the ALPS index and the MMSE score, and this might be due to the sensitivity of the MoCA-J score, which is greater than that of the MMSE score, to cognitive decline detection (45).

This study has some limitations. First, the study participants were only men. Meanwhile, female mice and rats showed a similar or more severe glymphatic dysfunction induced by RHI than males (14, 46). It is possible that the glymphatic system may be similarly impaired in women who have experienced RHI. Therefore, future studies are needed to investigate this possibility. Second, some confounding factors that lead to cognitive and glymphatic impairment were not considered owing to the time lag between the experience of contact sport and image acquisition. Although there have been no studies on RHI-related long-term brain pathological in humans, some animal studies have reported long-term gliosis and pathological changes in cerebral white matter after RHIs (9, 47), supporting its chronic effect on the glymphatic system. Third, previous studies have shown the associations between blood pressure and HbA1c and glymphatic system function (27). Therefore, we matched blood pressure among groups to minimize the effects of vascular risk factors in this study. In this study, HbA1c was not significant but tended to be higher in the noncontact group than that in the heavy-contact group, and diabetes mellitus is known to decrease glymphatic function (28, 48). Nevertheless, a significant difference in the left ALPS index was consistently observed after HbA1c was included as a confounding factor. The subjects in the noncontact group had a greater carbohydrate intake than the subjects in the other groups, which is the reason for the higher trend in HbA1c values (49). Furthermore, even when HbA1c was included as a covariate, the ALPS index tended to be lower in the semicontact and heavy-contact groups, suggesting that HbA1c had limited influence on the study's validity. Fourth, the ROIs for calculating ALPS indices include not only the medullary veins but also the surrounding cerebral white matter. Thus, it is impossible to exclusively evaluate the diffusivity of the perivascular space along the medullary veins. However, a strong correlation between the ALPS index and the functional assessment of the glymphatic system by intrathecal injection of gadolinium contrast agents *in vivo* was reported, which supports the use of the ALPS index (16). Fifth, our cohort does not review the history of mild traumatic brain injuries (concussive head impacts). However, according to the trauma history of all the subjects, they have not experienced severe traumatic brain injuries requiring hospitalization. Although the sole effect of mild traumatic brain injury cannot be considered, this study analyzed the effects of RHIs, including mild traumatic brain injuries and subconcussive head impacts, excluding severe traumatic brain injury. Finally, this study lacked histopathological validation, and no direct evidence supports the changes in AQP4 expression.

## 5. Conclusion

In summary, exposure to contact sports in youth may cause glymphatic dysfunction in old age. Furthermore, contact-sports-related glymphatic dysfunction is associated with cognitive decline.

## Data availability statement

The original contributions presented in the study are included in the article/supplementary material, further inquiries can be directed to the corresponding author.

## Ethics statement

The studies involving human participants were reviewed and approved by Juntendo University Hospital Ethics Committee. The patients/participants provided their written informed consent to participate in this study.

## Author contributions

YM, KK, CA, KT, JK, SF, TS, MM, TT, and SN contributed to the concept, design, and analysis of the study and drafted and revised the manuscript. WU, YSa, HT, HN, HK, YSo, YT, HW, and RK performed data and image acquisition, analyzed and interpreted the data, and revised the manuscript for intellectual content. TA, AW, OA, and SA supervised analysis, participated in study design, data interpretation, and drafted and revised the manuscript. All authors contributed to the study and approved the final manuscript.
